# Endoscopic ultrasound–guided gastroenterostomy for managing gastroparesis refractory to gastric peroral endoscopic pylorotomy: a promising new therapeutic option

**DOI:** 10.1016/j.igie.2025.01.011

**Published:** 2025-01-17

**Authors:** Jean-Michel Gonzalez, Juliette Phelip, Mohamed Gasmi, Véronique Vitton, Marc Barthet

**Affiliations:** Department of Gastroenterology, AP-HM, Hôpital Nord, Aix-Marseille Université, Marseille, France

## Abstract

**Background and Aims:**

Gastric peroral endoscopic myotomy (G-POEM) reaches an efficacy rate around 65% for gastroparesis. Endoscopic ultrasound (EUS)–guided gastroenteroanastomosis (EUS-GEA) has demonstrated efficacy in gastric outlet obstruction. We evaluated it for refractory gastroparesis.

**Methods:**

We undertook a single-center retrospective study enrolling patients with gastroparesis treated with G-POEM with failure or symptoms recurrence managed with EUS-GEA. The drain-assisted EUS-GEA technique with 20-mm lumen-apposing stent was applied. End points were clinical efficacy at 6 months, adverse events, and recurrence rate.

**Results:**

Twelve patients were included, median age 46 years (interquartile range [IQR], 16-78 y). Patients had an abnormal gastric emptying or bezoar, and 50% never improved from baseline. Median follow-up was 11.5 months (IQR, 6-26 mo). Clinical efficacy rate was 75% at 6 months. The median preoperative Gastroparesis Cardinal Symptoms Index was 4.1 (IQR, 1.6-5) versus 1.6 (IQR, 0-2.6; *P* < .05). No severe periprocedural adverse events occurred.

**Conclusions:**

EUS-GEA demonstrated promising results in improving patients with gastroparesis refractory to G-POEM. Prospective evaluation is required to confirm these results.

Gastroparesis is a gastric motility disorder with a prevalence of 0.1% in the population that can lead to altered quality of life.^1^ Its severity is evaluated using the Gastroparesis Cardinal Symptoms Index (GCSI). The physiopathology remains complex, associating gastric body hypomotility, antropyloric hyperpressure, and a reduced pyloric compliance.^2^ Among interventional procedures, gastric electrical stimulation has been proposed, surgically implementing the Entera system (Enterra Therapy, St Louis Park, Minn, USA), but with an efficacy limited to patients with chronic vomiting.^3^ For these reasons, interventional therapies targeting the pylorus have been developed, such as pylorotomy through gastric peroral endoscopic myotomy and intrapyloric injections of botulinum toxin.

To date, gastric peroral endoscopic myotomy (G-POEM) is one of the best options for treating refractory gastroparesis. Indeed, recent literature review demonstrated an efficacy rate between 56% and 80%.^4^, ^5^, ^6^ In the meantime, the procedure is safe, with fewer than 0.5% with severe adverse events reported in 2 large multicenter series.^7^ Based on these findings, the recent European Society of Gastrointestinal Endoscopy (ESGE) guidelines recommend G-POEM for treating severe and refractory gastroparesis in expert centers.^8^ Surgery such as sleeve gastrectomy and Roux-en-Y bypass has been proposed as well, but comes with inconsistent efficacy and the risk of fistula.^9^^,^^10^

As for endoscopic ultrasound (EUS)–guided gastroenteroanastomosis (EUS-GEA), the first procedure was realized by our team in 2014.^11^ Since then, the technique expanded especially for treating malignant gastric outlet obstructions (GOOs) for palliative purpose. This approach demonstrated technical and clinical success rates of 95% and 90%, respectively.^12^^,^^13^ Moreover, studies have demonstrated the superiority of EUS-GEA compared with duodenal stenting in terms of clinical efficacy and reintervention rate, and noninferiority compared with surgical GEA, but with a shorter hospital stay and a quicker time for resuming normal diet and chemotherapy.^12^ Therefore, ESGE recommends the use of EUS-GEA for the palliative management of GOOs, but remains careful with benign indications, because of a nonnegligible adverse event rate due to stent misdeployment. Despite that, an increasing number of cases are published concerning benign disease with promising outcomes. The nasobiliary drain–assisted technique (or WEST technique) is currently the best technique and allows for securing the procedure by reducing the risk of misdeployment.^13^

Therefore, we undertook a preliminary clinical and technical evaluation of EUS-GEA for managing gastroparesis refractory to G-POEM.

## Methods

### Design and eligibility criteria

This was a preliminary retrospective report of consecutive patients managed in our tertiary center from September 2021 to November 2022. The patients included had severe and disabling refractory gastroparesis, proven by gastric emptying scintigraphy (GES) that showed a delayed gastric emptying with more than 10% of gastric retention at 4 hours. All had undergone G-POEM and were not responders or experienced symptomatic relapse. The nonresponse was confirmed by absence of improvement greater than 1 point from baseline GCSI (before G-POEM) and GES findings showing a persistence of delayed gastric emptying at 3-month postoperative assessment. Finally, all underwent EUS-GEA as last-line therapy for their refractory disease.

All the cases were discussed in functional disease multidisciplinary meetings, which included surgeons, to validate the EUS-GEA. Surgical gastrojejunostomy was not proposed in such clinical situations, where it is known to be nonfunctional, and it is not part of our therapeutic algorithm. Laparoscopic sleeve gastrectomy was discussed in 2 cases, but the patients refused any open surgery. The modalities and the risks were clearly explained to the patients according to the current literature. Our database was anonymized and declared, according to the French law for retrospective studies, to the Comission Nationale pour la Liberté Informatique.

### Procedure

All procedures were performed by 3 endoscopists considered to be experts in therapeutic EUS, who had already performed more than 40 EUS-GEAs for malignant gastric outlet obstructions. Before the procedure, patients followed a liquid diet for 48 hours to ensure the absence of solid food in their stomach, which would contraindicate the procedure. The procedures were performed with the patient under general anesthesia, with orotracheal intubation, in supine position, and using therapeutic linear echoendoscopes with 4-mm operating channel (Pentax, Tokyo, Japan, or Fuji, Tokyo, Japan).

**Figure 1:**
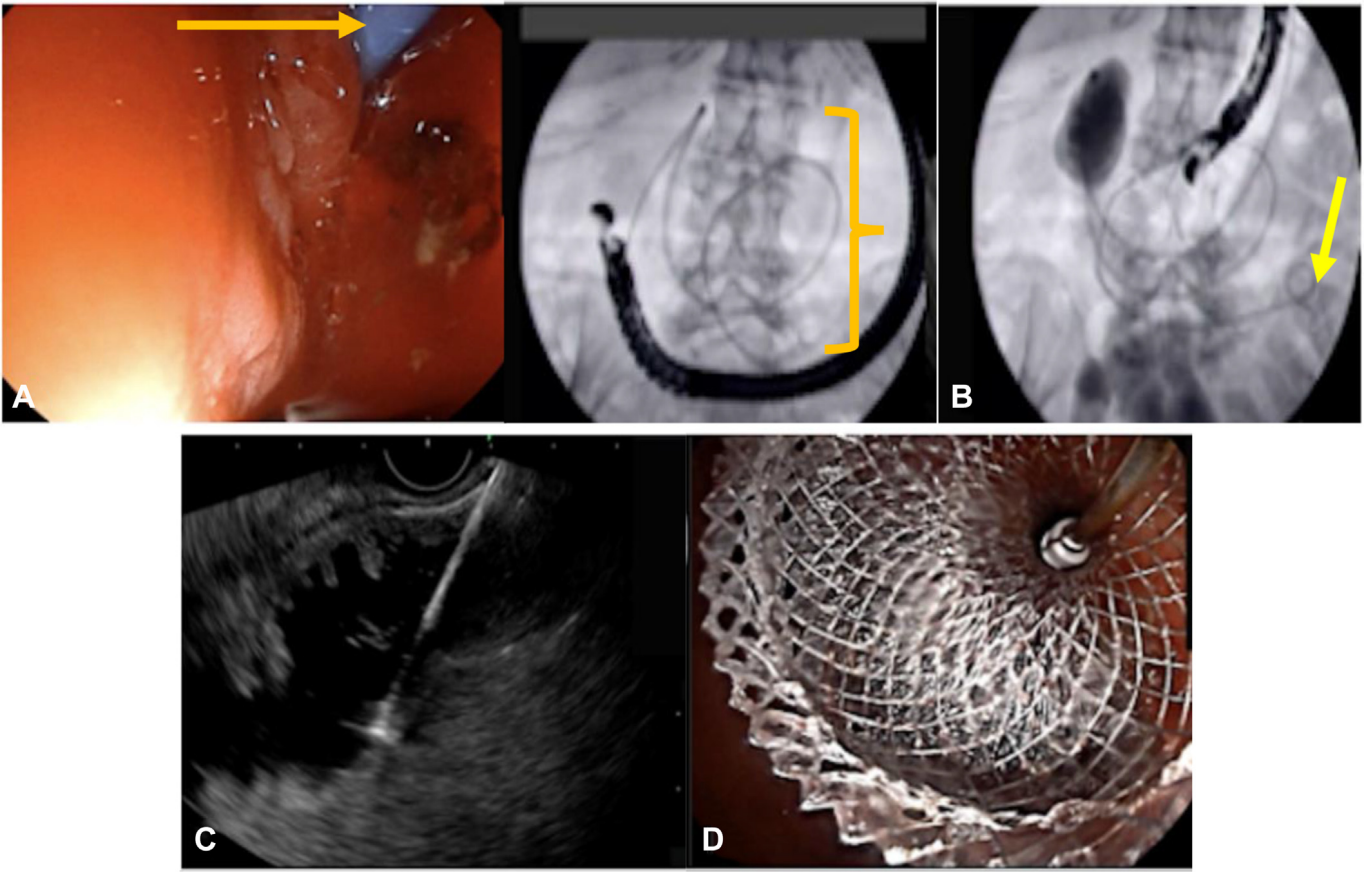
Procedural steps of the drain-assisted endoscopic ultrasound (EUS)–guided gastroenteroanastomosis. **A,** Placement of nasocavitary simple pigtail drain (see *arrow* and *bracket*) over a guidewire, advanced to duodenojejunal angle under fluoroscopic guidance. **B,** Filling of the jejunal limb with saline solution mixed with contrast (see *arrow*), **C,** Direct puncture of the jejunal limb with the electrocautery tip of the lumen-apposing metal stent (LAMS) applying pure cut current (effect 4.5), followed by the deployment of the distal flange of the LAMS into the jejunal lumen under EUS control. **D,** Deployment of the proximal flange of the LAMS into the gastric lumen under endoscopic control.

The EUS-GEA technique applied was the WEST technique,^14^ with the following steps (Fig. 1): (1) placement of a guidewire advanced up to the Treitz angle with the use of a double-lumen straight catheter (GT2T; Olympus, Tokyo, Japan); (2) placement of a nasobiliary drain at the level of the Treitz angle, secondarily plugged to an irrigation pump; (3) placement of the EUS scope into the stomach and detection of the jejunum, which is filled with saline solution mixed with contrast (Visipaque, GE Healthcare, Chicago, Ill, USA; 270 mg iodine/mL); (4) after injection of glucagon (2 times 2 mg Glucagen, Novo Nordisk Limited, Gatwick, West Sussex, UK), access to the distended jejunal lumen by direct puncture with the hot lumen-apposing stent (LAMS; Hot Axios 20 mm; Malborough, Mass, USA), applying pure cut current with effect 4.5 (electrosurgical unit VIO-3; Erbe, Marietta, Ga, USA), preloaded with a guidewire (in case a rescue procedure is necessary); (5) deployment of the distal flange under both EUS and fluoroscopic control; (6) deployment of the proximal flange under endoscopic control; and (7) drain retrieval and final contrast injection to confirm the good position of the LAMS.

Postoperatively, patients were kept fasting overnight with intravenous antiemetics. In the absence of adverse events, they resumed liquids on postoperative day (POD) 1 and then mixed diet from the evening of POD 1 and for 1 week. They were discharged at POD 2 to 3.

### End points and follow-up

**Figure 2:**
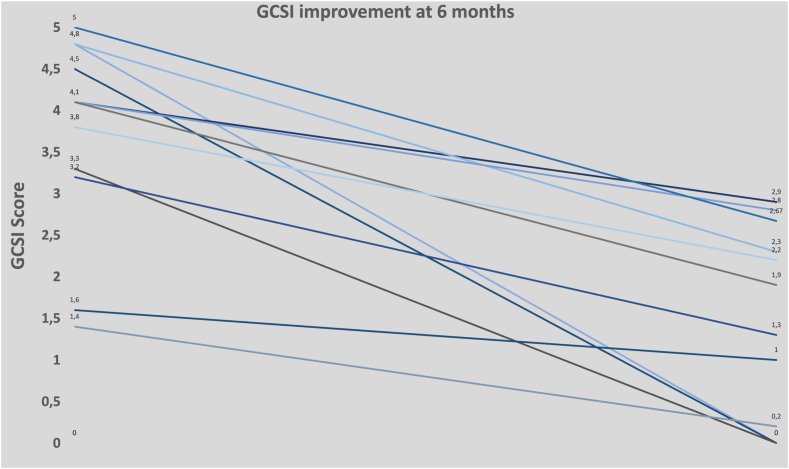
Graph showing the Gastroparesis Cardinal Symptoms Index (GCSI) score evolution at 6 months compared with preoperatively for each patient.

Patients were followed at 3 and 6 months and then annually by clinical evaluation including GCSI calculation. The primary objective was to document the clinical efficacy rate, defined as decrease of GCSI of more than 50% compared with baseline. A partial efficacy was defined as a decrease greater than 30% or 1 point from baseline. The secondary objectives were to document the weight evolution and the security profile of the procedure assessed according to the AGREE classification, which is the validated European score to assess adverse events in digestive endoscopy (Fig. 2).^14^

## Results

### Patient’s characteristics

Eighteen patients were eligible because they underwent EUS-GEA for gastroparesis refractory to other therapies. Three of them were excluded because they had undergone surgical gastrojejunostomy, which was nonfunctional, and 3 others because their follow-up was less than 6 months. Twelve patients were finally included, aged 20 to 78 years, with a median follow-up of 11.5 months.^6^, ^7^, ^8^, ^9^, ^10^, ^11^, ^12^, ^13^, ^14^ The origin of gastroparesis was idiopathic in 9 cases and diabetic in 3.

After the initial G-POEM, 7 patients (58%) were nonresponders at 3 months. Five patients (42%) had symptoms relapse after a median of 20.5 months (interquartile range [IQR], 12-48 mo). All of them had prescriptions of prokinetics such as erythromycin and domperidone, 1 had gastric electrical stimulation, and 1 had a percutaneous gastrostomy, all without success. The median time between G-POEM and EUS-GEA was 19 months (IQR, 5-121 mo). Their median GCSI score before GEA was 4.1 (IQR, 1.6–5). The mean H4 retention was 29% ± 33% (Table 1).

**Table 1:** Median (interquartile range) total GCSI and subscale scores at baseline and at 6-month follow-up

	Preoperative	6-month postoperative
**Vomiting subscale**	3.65 (1-5)	0.3 (0-2)
**Satiety subscale**	4.75 (3.25-5)	1.75 (0-3.25)
**Bloating subscale**	4.5 (0-5)	1.25 (0-5)
**Total GCSI**	4.1 (1.4-5)	1.6 (0-2.9)

*GCSI*, Gastroparesis Cardinal Symptoms Index.

### Technical outcomes

The procedures of EUS-GEA were uneventful, without stent misdeployment. The postoperative course was uneventful for all patients except 1, with a median hospital stay of 2 days.

The last patient had gastrointestinal bleeding with melena and blood loss requiring transfusion of 2 units of blood cells. The upper-gastrointestinal endoscopy did not find any lesion and there was no recurrence, so the patient was discharged at POD 6. This adverse event is classified as mild, AGREE IIIa.

Four patients had systematic control of the GEA at 6 months with the aim to check for tissue overgrowth or local adverse events such as ulcers. The LAMSs were in place and intact. A small asymptomatic ulceration of the small bowel was detected in 2 patients who were preventively administrated oral proton pomp inhibitors (20 mg daily). Two patients had a follow-up examination at 1 year, and the LAMSs were still in good position, without any ingrowth or structural alteration.

### Clinical outcomes

A complete clinical efficacy was observed in 75% of the patients (9/12), with a GCSI score at 0 in 4 of them. Regarding the 3 remaining patients, they had partial efficacy with GCSI decreasing between 30% and 50%. The median postoperative GCSI score was significantly decreased compared with the baseline: 1.6 (0-2.6) versus 4.1 (1.4-5) (*P* < .06). All patients stabilized or gained weight after the intervention.

The median follow-up was 11.5 months.^6^, ^7^, ^8^, ^9^, ^10^, ^11^, ^12^, ^13^, ^14^ Two patients experienced partial symptoms recurrence: one at 7 months, but surprisingly had a normal barium swallow examination and no gastric residual; and the other one after 14 months, but was diagnosed with chronic intestinal pseudo-obstruction.

## Discussion

Gastroparesis is a complex disease with an incompletely known physiopathology. This probably explains why many treatments remain disappointing. G-POEM recently brought hope to these patients, coming with safe and quite effective outcomes, but one-third of the patients do not sustain mid-term clinical response to this therapy. However, in such situations, there is no other therapeutic alternative and surgery may be considered, but with a lack of evidence.

In the meantime, EUS-GEA was recently developed for malignant gastric outlet obstructions with excellent and competitive outcomes compared with surgical GEA. Moreover, in the most recent series, benign indications are expanding, reaching almost 30% of GEA procedures, and the drain-assisted approach has decreased the adverse event rate related to stent misdeployment.^13^

Therefore, we present the first preliminary series assessing the outcomes of drain-assisted GEA for managing gastroparesis refractory to all therapies. The patients also had severe diseases, with a mean GCSI >3, and sometimes weight loss requiring parenteral nutrition. Despite considering the retrospective design, the outcomes are impressive, showing a clinical improvement in 75% of the patients, even applying a strict definition of success. Interestingly, 4 patients had a complete disappearance of symptoms (GCSI = 0). Moreover, using the drain-assisted technique for EUS-GEA, the procedures were uneventful, and only 1 mild to severe adverse event occurred (bleeding) and was managed conservatively with no consequences.

One explanation could be that after G-POEM, the food needs to reach the pylorus, which may not be the case with gastric body hypomotility, whereas GEA offers a second exit at the junction of the body and antrum.

In conclusion, EUS-GEA seems safe and a new effective therapeutic line for treating refractory gastroparesis. These preliminary results must obviously be confirmed in prospective cohorts.

## Disclosure

The following authors disclosed financial relationships: J.M. Gonzalez: consultant for Boston Scientific, Fujifilm, and Pentax. M. Barthet: consultant for Boston Scientific, and Fuji. All other authors disclosed no financial relationships.
